# Effect of diabetes and hyperglycaemia on the physical and mechanical properties of dentine: a systematic review

**DOI:** 10.1007/s00784-025-06151-5

**Published:** 2025-01-10

**Authors:** Kuan-Chieh Hwang, Joanne Jung Eun Choi, Haizal Mohd Hussaini, Paul R. Cooper, Lara T. Friedlander

**Affiliations:** Sir John Walsh Research Institute, Faculty of Dentistry, University of Otago, Dunedin, New Zealand

**Keywords:** Diabetes, Mechanical properties, Dentine, Hyperglycaemia, Systematic review

## Abstract

**Objectives:**

The aim of this systematic review was to assess the effect of DM (Type 1 and Type 2 Diabetes) and hyperglycaemia on the physical and mechanical properties of dentine which is critical for successful endodontic treatment.

**Method:**

An electronic search of the following databases: PubMed, MEDLINE, Web of Science and the grey literature was performed up until July 2024. In vitro and in vivo studies on the effect of DM or hyperglycaemia on the mechanical and physical properties of dentine were included. Non-English language literature was excluded.

**Results:**

Of the 234 articles identified, 15 met the inclusion criteria. Four studies evaluated how artificially induced glycation or natural glycation of dentine due to aging affects the mechanical properties of dentine. Five studies investigated the influence of Type 2 Diabetes (T2D) on dentine’s mechanical properties, while two studies focused on the effects of Type 1 Diabetes (T1D). A further, four studies compared the effects of both T1D and T2D on the dentine. The studies were heterogeneous and a range of mechanical and physical properties were evaluated.

**Conclusion:**

DM and AGEs negatively influence the physical and mechanical properties of dentine however, there remains a paucity of evidence and further studies are needed.

**Clinical significance:**

Diabetes Mellitus (DM) is a chronic metabolic disease characterised by hyperglycaemia, an altered immune response and complications associated with collagen connective tissues. DM can influence bone metabolism and alter its physical and mechanical properties via glycation processes within collagen and changes to osteoblast activities. While bone and dentine share similarities, dentine is unique as it is intimately associated with the dental pulp. Inflammation within the pulp can induce calcification and tertiary dentine deposition and so exploring the influence of DM on the mechanical properties of dentine is warranted to understand the clinical significance.

## Introduction

Diabetes mellitus (DM) is a chronic metabolic disease characterised by hyperglycaemia, an altered immune response, micro- and macro-vascular changes, and complications associated with connective tissue and wound healing. It is frequently associated with obesity and a range of co-morbidities [1]. There are three common types of diabetes. Type 1 diabetes (T1D) which is caused by the autoimmune destruction of beta pancreatic cells resulting in lack of insulin production. The onset of T1D commonly occurs during childhood or adolescence [2] and gestational diabetes which occurs during pregnancy and is a risk factor for developing Type 2 diabetes (T2D) [3]. T2D is a consequence of impaired insulin secretion by the beta pancreatic cells and/or impaired insulin function within the peripheral tissues [4]. T2D is the most common and typically develops later in life due to genetic and lifestyle factors including an unhealthy diet and physical inactivity [5].

The International Diabetes Federation estimated that 537 million individuals were living with diabetes in 2021, making up 10.5% of the global population, and it estimated that the number of people affected by diabetes will increase to 643 million people by 2030 and 783 million people by 2045 [6]. Since T2D represents 81–92% of all diabetes cases, its prevalence is projected to increase by 20% in developed countries and by 69% in developing countries between 2010 and 2030 [7, 8].

Glycation is an intra- or extra-cellular biological process involving the modification of lipids or proteins following exposure to carbohydrates and is associated with physiological aging and the pathogenesis of complications associated with T2D. This non-enzymatic process leads to the formation of non-reversible products known as advanced glycation end-products (AGEs). AGEs may accumulate in permanent and long-lived proteins, such as collagen leading to AGE-collagen cross-links (Maillard reaction) and fibrosis of collagen-rich tissues. Collagen is essential for mechanical function [9], however AGEs can accumulate in bone collagen, restricting molecular sliding and increasing brittleness [9]. AGEs also inhibit osteoblast activity [10–12]. In patients with diabetes and this leads to impaired mineralization and lower bone turnover [10].

Patients with T1D have low bone mineral density while those with T2D tend towards high bone mineral density and are at up to three times greater risk of fractures [13]. The exact mechanisms for these differences are not fully appreciated but it has been suggested that the accumulation of microcracks and/or increased cortical porosity may contribute [13]. Notably, hyperglycaemia can affect osteoblast mineralization by downregulating the expression of alkaline phosphatase [14]. Reports from animal studies have shown the negative effects of T2D on bone biomechanical indices, including energy to yield, energy to break, and toughness. Specifically, T2D diminishes the bone’s capacity to absorb energy when it is subjected to bending forces [15, 16].

New evidence is emerging that indicates glycation processes may also exert an influence on the dentine-pulp complex. The dentine and pulp are embryologically and morphologically related. Most recently, laboratory studies have shown that the pulp tissue from patients with T2D has an increased base-line inflammatory load as well as a greater density of collagen and proteins for AGEs within the connective tissue. Pulp calcifications, both free and attached to the walls of the pulp chamber, are also frequently observed compared to patients without disease [17–19]. Patients with T2D have an increased risk of vertical root fracture [20], however the reason for this is unclear. Furthermore, glucose oxidative stress in rats has been shown to downregulate the expression of dentinogenesis-related proteins, including BMP-2, BMP-7, DSPP and DMP1, and development of a thickened layer of predentine [18, 21].

Given diabetes affects collagen synthesis and is associated with calcification within the pulp, it is reasonable to propose that hyperglycaemia may also influence the mechanical properties of dentine however the current understanding of this is limited. Notably, the process of dentinogenesis shares similarities with bone formation. Unmineralised dentine (predentine) is secreted by odontoblasts throughout their lifespan. This zone is characterized by a collagen meshwork, primarily composed of Type I collagen. Collagen is exocytosed near the odontoblast cell bodies and cross-links as it progresses toward the mineralization front. At the predentine-dentine junction, odontoblasts secrete intertubular dentine and non-collagenous proteins including proteoglycans, which contribute to the formation of intertubular dentine [22]. Peritubular dentine is secreted at a distance from the predentine and is formed by deposits of amorphous matrix along the lumen of the dentinal tubules [22]. Factors such as the quantity of intertubular and peritubular dentine, the location, orientation, and density of dentinal tubules, the direction of collagen fibres, and mineral densities all influence the mechanical properties of dentine [23]. Given healthy collagen and patent tubules are necessary for optimal dentine bonding, and brittle dentine is more prone to fracture understanding the influence of hyperglycaemia on the behaviour of dentine is especially relevant in the clinical environment.

The aim of this review was to systematically synthesise data and available evidence related to the effects of hyperglycemia and T1D and T2D on the physical and mechanical properties of dentine as they are critical in ensuring successful endodontic treatments.

## Methods

This research adheres to the guidelines set out by the Preferred Reporting Items for Systematic Reviews and Meta-Analyses (PRISMA) framework [24].

### Inclusion and exclusion criteria

The inclusion criteria included: (1) Study articles published in standard English language; (2) Articles that match an online database search using the keywords of selection; (3) In vitro and in vivo studies that investigated the effects of T1D, T2D and AGEs on the physical and mechanical properties of dentine. Publications that did not meet these criteria were excluded.

### Search methodology

A systematic electronic search of articles was performed using PubMed [1964–2024], OVID Medline [1946–2024] and Clarivate Analytics’ Web of Science [1980–2024] up to April 2024. In addition, manual searches in the reference list of the included records was also conducted. The search methodology employed a combination of keywords and Medical Subject Heading (MeSH) terms, utilizing the Boolean operators ‘AND’ and ‘OR’ to refine the results (Table 1). Only English language literature was included and no time (i.e. year of publication) restriction was applied. The search was conducted up to the end of July 2024. The PICO model was assigned for the search; P: dentine; I: Type 1 or 2 Diabetes Mellitus or hyperglycaemia; C: No Type 1 or 2 Diabetes Mellitus or hyperglycaemia; O: Physical and mechanical properties.

**Table 1:** Key words used in the search

**Pubmed**	((Diabetes OR “type 2 Diabetes” OR “Type 1 Diabetes” OR Hyperglycaemia OR “Advanced glycation end product*” OR “advanced glycation end-products”) AND (Dentin*)) AND (“Shear bond strength” OR “tensile bond strength” OR “mineral density” OR microhardness OR fracture OR “bond strength”)
**Ovid MEDLINE**	1-(“Diabetes Mellitus” OR “Type 2 diabetes” OR “Type 1 diabetes” OR “Hyperglycaemia”).mp. [mp = title, book title, abstract, original title, name of substance word, subject heading word, floating sub-heading word, keyword heading word, organism supplementary concept word, protocol supplementary concept word, rare disease supplementary concept word, unique identifier, synonyms, population supplementary concept word, anatomy supplementary concept word] 2-exp dentin/ OR exp dentin, secondary 3-(“mechanical propert*” OR “Shear bond strength” OR “tensile bond strength” OR “mineral density” OR microhardness OR fracture OR “bond strength”).mp. [mp = title, book title, abstract, original title, name of substance word, subject heading word, floating sub-heading word, keyword heading word, organism supplementary concept word, protocol supplementary concept word, rare disease supplementary concept word, unique identifier, synonyms, population supplementary concept word, anatomy supplementary concept word] 4-1 AND 2 AND 3
**Web of science**	TS=(((Diabetes OR “type 2 Diabetes” OR Type 1 Diabetes” OR Hyperglycaemia OR “Advanced glycation end product*” OR “advanced glycation end-products”) AND (Dentin*)) AND (“Shear bond strength” OR “tensile bond strength” OR “mineral density” OR microhardness OR fracture OR “bond strength”))

### Study selection

Three independent investigators conducted the study selection process in two phases. During the initial phase, one researcher (KCH) reviewed the titles and abstracts of studies identified through the search. Search results were imported into EndNote (version X9, Clarivate, London, UK) to identify and eliminate any duplicate entries. A full-text review of the potential studies for inclusion was conducted to evaluate their eligibility. Two reviewers (L.F. and J.C.) discussed and resolved any uncertainties regarding a study’s eligibility. The relevant study characteristics were exported to Microsoft Excel software (Version 16.90, Microsoft). Studies with titles and abstracts meeting the eligibility criteria were selected. When titles and abstracts provided insufficient information for decision-making, the full texts were obtained for further scrutiny.

In the second phase, the researchers (K.C.H, J.C. and L.F.) performed a comprehensive assessment of the selected full-text articles. Studies that fully met the eligibility criteria upon full-text review were included in the final selection. Any disagreements between the three investigators were resolved through discussion.

### Data collection and analyses

A single researcher extracted key information from selected studies including: the last name of the first author, publication year, study design, groups involved, sample size, objectives, and type of analysis, evaluation methods and key findings. The data extraction process utilized a pre-tested form in an Excel spreadsheet.

Following this initial extraction, co-researchers (J. C and L. F) conducted a review of the collected data to ensure accuracy and completeness. Disagreements were resolved through consensus or by discussing with the co-researchers.

### Risk of bias assessment

Three independent reviewers (K. C. H, J. C., and L. F.) evaluated the risk of bias (RoB) in the included studies. For animal studies, an adapted version of the SYRCLE’s RoB risk of bias tool was used [25]. This incorporated an assessment of sample size justification to provide a more comprehensive evaluation of the reporting quality in these studies. To ensure a systematic and standardized evaluation of the methodological quality and potential biases, reviewers used discrete criteria: A ‘no’ judgement indicated a high risk of bias, a ‘yes’ judgement indicated a low risk of bias, An ‘unclear’ judgement was given when there was insufficient information or uncertainty regarding the potential for bias.

For in vitro studies, each study was evaluated for risk of bias using a modified version of the Consolidated Standards of Reporting Trials (CONSORT) [26]. Since this systematic review included only in vitro studies, items relevant to clinical studies were excluded from the assessment. For each applicable item, a ‘Yes’ or ‘No’ determination was made based on the item description. Two reviewers (J.C. and L.F.) cross-checked the risk of bias assessments.

Each study received an overall risk of bias score and was categorized as follows: Low (there is a low concern that bias could affect the study results), moderate (there is a likely risk of bias, with some reason to doubt the results) and high (there is a high likelihood of bias, with considerable reason to believe it may alter the results).

### Synthesis of results

A narrative synthesis of results was undertaken recognising that there was heterogeneity between groups and assessment methods across studies. Furthermore, some studies failed to report essential statistical analyses, such as standard deviations for mean differences.

## Results

### Studies selected for inclusion

**Fig. 1:**
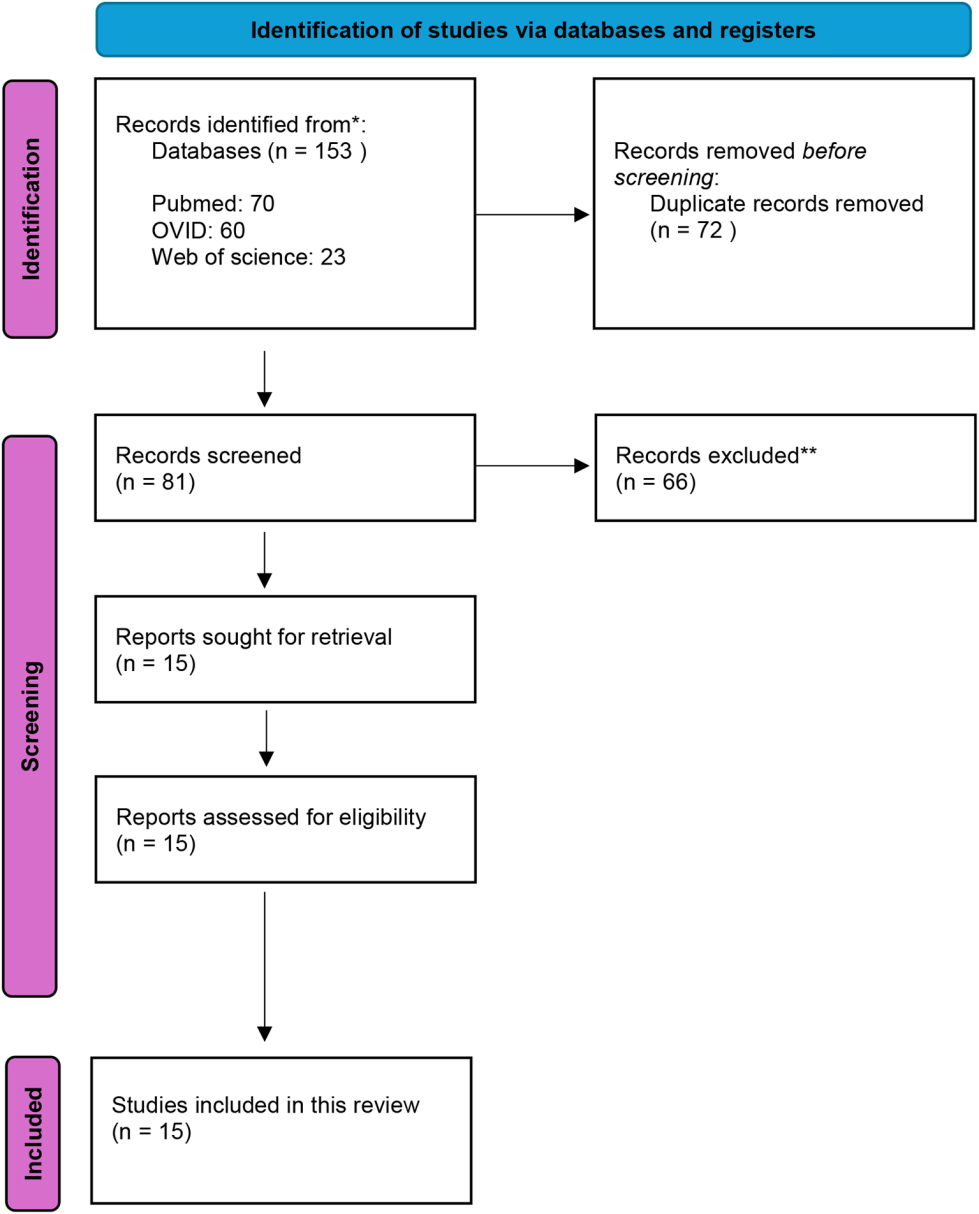
PRISMA 2020 flow diagram used in this study

The flowchart of the search process is shown in Fig. 1. A total of 234 articles were identified. After screening, duplicates and records without full texts were removed and 15 studies met the inclusion criteria and were qualitatively analysed [27–41]. Four studies examined how artificially induced glycation or natural glycation of dentine due to aging affects its mechanical properties [28, 29, 39, 40]. Five studies solely focused on investigating the influence of T2D on the mechanical properties of dentine [31, 33–35, 37], while two studies considered the effects of T1D [27, 31]. A further four studies investigated and compared the effects of both T1D and T2D on the mechanical properties of dentine [32, 36, 38, 41].

### Characteristics of the included studies

According to the study design, two studies were in vivo, and 13 studies were in vitro (Table 2). Of the two in vivo studies, Abbassy et al. (2015) evaluated the effect of T1D on mineral density and morphological changes of enamel and dentine in male Wistar rats, and Saghiri et al. (2022) assessed the effect of T1D on the microhardness of enamel and dentine in C57BL/6J mice. Of the remaining 13 in vitro studies, they evaluated various mechanical and physical properties using extracted teeth from individuals with T1D or T2D, or glycated normal human teeth [28–30, 32–41]. Two studies evaluated the levels of trace elements in T2D teeth and its effect on dentine structure, tubular density, root fracture resistance and microhardness [34, 35]. One study evaluated the relationship between tubular density and the push-out bond strength of mineral trioxide aggregate (MTA) to T2D dentine [33]. A further study investigated the effects of sodium hypochlorite and EDTA on the microhardness and erosion susceptibility of T2D dentine [30]. Three studies were conducted to investigate the tensile and shear bond strength (SBS) of composite to both T1D and T2D dentine [36, 38, 41]. Additionally, they also investigated the effect of diabetes on fracture resistance of teeth obturated with different root fillings on extracted T2D teeth [37] and the effect rotary instrumentation on both T1D and T2D dentine [30, 32]. Four in vitro studies investigated the effect of induced glycation on dentine, rather than the direct effects of diabetes on dentine. Miura et al. (2013) evaluated whether advanced glycation end products (AGEs) accumulate in dentinal collagen and how this may affect the hardness of dentine. Shinno et al. (2016) also investigated how AGEs diminish the strength of aged dentine. Haluszka et al. (2022) evaluated the impact of glycation on dentinal collagen, dentine structure and mechanical properties [28]. Alania et al. (2020) investigated the effect of induced glycation on the ultimate tensile strength, energy to fracture and elastic modulus of dentine [40].

**Table 2:** Reported outcomes of included studies

Articles	Aim	Sample/sample size	Outcome measured	Results
Shinno et al., (2016).	To investigate the mechanical properties of human dentine in terms of mineral density and structural and quality parameters.	Freshly extracted human molars (*n* = 46) sectioned into beam-shaped crown and root dentine, and semicircular shaped crown dentine obtained.	Mineral density. Flexural strength and toughness. Dentinal tubule density and degree of occluded tubule lumens. Hardness and Youngs modulus. Analysis of apatite orientation. Quantification of AGEs.	Aged dentine showed a high AGEs level in its collagen and low flexural strength. Dentine with a high density of dentinal tubules tended to have low flexural strength. AGEs content - A significant negative correlation was found between the flexural strength and the pentosidine content of both crown and root dentine.
Saghiri, Nath, et al. (2021).	The root fracture resistance (RFR) of premolars extracted from diabetic patients and the effect of biomaterials: white mineral trioxide aggregate (WMTA) and WMTA + Na2HPO4 as an additive, on enhancing RFR were evaluated	Single canal premolars from T2D and non-diabetic teeth (age 20–60 y/o). 4 subgroups (*n* = 5 each) 1. RCT obturated with WMTA 2. RCT obturated with WMTA + NA2HPO4 3. Gutta-percha 4. unfilled (control)	RFR analysis	The mean RFR values of T2D specimens were significantly lower. In the non-diabetic group - Lowest RFR was found in the control subgroup and highest RFR in the WMTA subgroup. In the T2D group - lowest RFR was found in the control subgroup and highest RFR in the WMTA + NAxHPO4 subgroup. The RFR in the diabetic patients was significantly lower, indicating their higher susceptibility to fracture under vertical forces. The use of WMTA (with or without Na2HPO4) for obturation enhances the RFR.
Saghiri, Saghiri et al. (2023)	To investigate the effect of inorganic trace elements - Mg, Sr and Zn on root canal dentine.	300 extracted human premolars (age20-60 y/o) from T2D and divide into 3 groups according to the solutions used (Mg, Sr, Zn). Each group subdivided int 5 subgroups according to the duration of the teeth soaked in each solution − 0 (control), 1, 2, 5, and 10 min. (*n* = 20 per subgroup).	RFR analysis. SuH (surface microhardness) of the roots analysed at 100- and 500-µm depths from the pulp-dentine interface. TD (Tubular density) analysis.	Tx with Mg, Sr and Zn significantly increased the RFR and SH, and decreased the TD in diabetic individuals
Saghiri, Freag, et al. (2021).	To evaluate the effect of diabetes mellitus (DM) on the tensile bond strength (TBS) of dental composite resin bonding to enamel and dentine of extracted human teeth.	30 caries free human premolar (age 20–60 y/o) from T1D, T2D, and non-diabetic. *N* = 10 in each group. Mid-root dentine was used.	Tensile bond strength (TBS) - using total etch technique and Universal bonding agent.	The TBS values were significantly higher in the control group compared to the T1D and T2D groups (*P* < 0.05). Between the DM groups, T2D values were significantly higher than those in the T1D group. Unlike DM groups, the zigzag fracture pattern was only noticed in the control group. DM adversely affected the TBS of dental composite resins to dentine; this negative effect is more exaggerated by T1D than T2D.
Saghiri et al. (2020).	Compared the tubular density and push-out bond strength of mineral trioxide aggregate (MTA) to dentine in diabetic and nondiabetic patients.	10 single rooted human teeth (age 20/60 y/o) from diabetic and non-diabetic patients (5 in each group). Mid-root dentine was used.	Push-out bond strength. Maximal load to dislodge was recorded in newtons. Specimens were viewed under a stereomicroscope (x21.25) and a SEM was used to determine failure types and tubular density.	Diabetic pts - significantly lower push-out bond strength of MTA to root canal dentine. The tubular density was significantly higher in diabetic patients. Failure type - mainly adhesive (4/5) in the healthy group. 3 adhesive, 2 structural failures in the diabetes group.
Miura et al. (2013)	To examine whether AGEs are formed in human dentinal collagen, and to consider any possible influence of AGEs on dentinal physiology.	6 caries-free 3rd molars of young and aged pts (18–26 yrs, 68–76 yrs). Demineralized dentine blocks were obtained. Some demineralized sections were glycated with 0.1 M ribose solution for 6 weeks.	Microhardness, fluorescence spectra and immunohistochemical analyses of demineralized dentine sections from young subjects were compared with those of aged ones. The same investigations were performed with young dentine artificially glycated by incubation in 0.1 M ribose solution.	The sections from aged dentine were mechanically harder than those from young dentine. The hardness of young dentine increased after incubation in ribose solution. Existence of AGEs in dentinal collagen was confirmed by immunohistochemical analysis. These changes were considered to be due to accumulation of AGEs. The obtained results suggest that AGEs accumulation occurs in dentinal collagen and is affected by both human age and physiological conditions such as glucose level in blood because dentinal collagen receives nourishment via dental pulp and tubules.
Saghiri, Rahmani, et al. (2022).	Microhardness, Erosion	48 single rooted maxillary and mandibular premolars (age 20–60 y/o): 24 T2D vs. 24 non-T2D. Mid-root 4 mm dentine slices were obtained. Specimens were assigned to four subgroups (*n* = 6) and irrigated for 5 min: (1) 2.6% sodium hypochlorite (NaOCl); (2) 17% ethylenediaminetetraacetic acid (EDTA); (3) 2% chlorhexidine (CHX); and (4) normal saline.	Surface microhardness was determined at 100- and 500-µm depths from the pulp–dentine interface. SEM was used to determine the severity of dentine erosion.	Diabetes as well as NaOCl and EDTA decreased surface microhardness of dentine significantly. Diabetes had little effect on the erosion susceptibility of dentine.
Saghiri, Sheibani et al. (2022).	Microhardness, tubular density and Sr and Mg levels.	35 T1D mice vs. 35 non-T1D mice. Each group subdivided into 7 groups of 5 different time points − 0, 1, 4, 8, 12, 20, and 28 weeks.	The microhardness was tested at 100- and 500 - µm depths from the pupal-dentine interface. Dentinal tubular density was determined by SEM and the levels of Sr and magnesium Mg was determined by color dot map analysis.	T1D negatively affected enamel and dentine microhardness, and enamel was influenced much more negatively and rapidly compared with dentine in diabetic groups. The dentinal tubule density in diabetic specimens was found to be lower compared with non-diabetic ones. Sr and Mg were reduced significantly in most subgroup.
Saghiri, Vakhonovetsky et al. (2023).	To evaluate the effect of DM on the nanostructure of root canal dentine	20 extracted human premolars (age 20–60 y/o): 10 non-diabetes vs. 10 T2D. Teeth sectioned into forty 2 mm thick dentine discs from coronal root third.	ICP-MS was used to determine the different elemental levels of copper, lithium, zinc, selenium, strontium, manganese, and magnesium. HRTEM was used to analyse the shape and quantity of the apatite crystals in diabetic and nondiabetic dentine at the nanostructural level.	Significant differences in trace element concentrations between the diabetic and nondiabetic specimens with lower levels of magnesium, zinc, strontium, lithium, manganese, and selenium, and higher levels of copper in diabetic specimens. Diabetic dentine exhibited a less compact structure with smaller crystallites and significantly more crystals in the 2500 nm2 area.
Abbassy et al. (2015).	To determine the mineral density and thickness of enamel and dentine	10 T1D rats vs. 10 non-T1D rats. Crown of lower incisor examined.	Mineral density and thickness of enamel and dentine was analysed.	The enamel and dentine thickness were significantly reduced (hypoplasia) and there was a significant reduction of the rate of dentine mineral apposition and formation, while there was no significant effect of the T1D condition on the mineral density of enamel and dentine.
Saghiri, Obeidi, et al. (2021)	To determine the effect of two types of diabetes on the shear bond strength of enamel and dentine, by using the single bond universal bonding system.	Sixty specimens from 15 teeth (age 20–60 y/o); 5 from each group—non-diabetic (ND), T1D, and T2D, were prepared with equal amounts of coronal dentine (*n* = 5) and enamel (*n* = 5).	Shear bond strength (SBS) tests - using total etch technique with Single Bond Universal Adhesive.	Enamel: significant difference between non-diabetic and T1D, and non-diabetic and T2D. No significant difference between T1D and T2D. Dentine: significant difference between non-diabetic and T1D. No significant difference between T1D and T2D, and Between Non-diabetic and T2D. Failure mode analysis: T1D - highest adhesive failures with dentine being the highest. 40% of enamel and 20% dentine showed cohesive failure. 60% of dentine showed adhesive. ND - showed mix of cohesive and mixed failures in enamel and dentine groups. 60% of enamel and dentine showed cohesive failures. 40% of enamel and 20% of dentine showed mixed failure. 20% of dentine showed adhesive failures. T2D - only dentin showed adhesive failure (20%), enamel showed highest cohesive failure of 60%. Mixed failure value of 60%.
Saghiri, Aminsobhani, et al., (2021).	To compare the amount of dentine removed by an endodontic rotary file, comparing dentine from diabetic patients with dentin from control patients under laboratory conditions	Non-diabetic vs. T1D vs. T2D *n* = 6 in each group. 18 mandibular incisors (pt age 20–60 y/o). 5 mm thick slices from mid-root section.	Depth of penetration by ProTaper F3 files. Dentine disc weight loss after intervention also measured.	Significantly more dentine was removed, and the penetration of the F3 instrument was significantly higher (P, 0.05) in DM specimens. T1D specimens having the highest mean penetration (approximately 3 times that of the normal specimens) and T2D specimens having approximately 2 times more mean penetration than that of the normal specimens. T1D specimens having a mean weight loss of approximately 5 times that of the normal teeth specimens and T2D specimens having approximately 2 times the mean weight loss compared with the normal specimens.
Haluszka et al., (2022).	Aimed at correlating the impact of glycation on the structure and mechanics of dental collagen by using multiphoton microscopy and AFM techniques	Non carious human molar teeth (*n* = 2) extracted from a single patient were used. Demineralized dentine sections were obtained, and some sections were artificially glycated in 0.5 M ribose solution for 10 weeks.	Multiphoton excitation fluorescence microscopy to assess the extent of glycation. The length of collagen fibers in intertubular dentine between tubules was measured by using ImageJ software. AFM imaging and nanomechanical tests - Young’s modulus and stiffness were determined.	Nanoindentation of intertubular dentine regions revealed significantly higher stiffness in the ribose-treated samples, which points at a significant accumulation of AGEs. Mean Young’s modulus of 36.62 ± 23.15 MPa was calculated for the ribose-treated specimens compared to controls, which showed an elastic modulus of 10.6 ± 4.71 MPa. In sum, glycation resulted in a significantly increased elastic modulus of dental collagen.
Attia et al., (2024)	To evaluate the influence of diabetes mellitus and the mode of applying a universal adhesive on the shear bond strength of composite resin to dentine.	40 molar teeth (age 20–60 y/o)- 20 = T1D, 20 = T2D, 20 = non-diabetic(ND) patients. Mid-coronal dentine was used. Each group also subdivided into 2 groups (10 each) - Universal adhesive total-etch vs. Universal adhesive self-etch.	SBS test with total etch or self-etch technique with Scotch Bond Universal. Bonding failure types determined using a stereomicroscope.	There was a statistically significant difference of Mean ± SD of shear bond strength among the three tested groups. In group ND, the Mean ± SD were (21.710 ± 0.638), it was decreased in group T1D to (14.626 ± 0.726) and group T2D to (17.740 ± 0.668). Total-etch subgroup had lower shear bond strength values than self-etch subgroup in all tested groups. The difference between self-etch and total-etch in each three groups was significant. ND specimens treated with universal adhesive in self-etch mode exhibited the highest incidence of cohesive failures. T1D group exhibited the greatest number of adhesive failures compared to the other two groups.
Alania et al., (2020).	the aim of this study was two-fold: (1) to determine an experimental model of accumulation of early glycation to assess the mechanical properties of root and crown dentine matrices and (2) to assess the in vitro inhibitory potential of aminoguanidine during the accumulation of early glycation of dentine.	Dentine Sect. (0.5 mm thickness, 6 mm length) from crown and roots of extracted human third molars (healthy patient). Specimens were exposed to three conditions - induced glycation, inhibition of glycation with aminoguanidine (AMG) and control, at three time periods (7, 14 and 21 days). Three mechanical outcome variables (*n* = 15 per group) - ultimate tensile strength, energy to fracture, apparent modulus of elasticity - were measured. *n* = 5 for the histological analysis. In vitro glycation of dentine specimens was induced by 0.6 M ribose solution.	Ultimate-tensile-strength, energy to fracture and apparent modulus-of-elasticity (*n* = 15 per group) were assessed. Histological imaging of collagen with picrosirius red staining assessed structural and conformation changes to dentine matrix. The UTS was expressed in MPa.	Ribose-induced glycation (Gly) resulted in statistically higher UTS when compared to control (*p* < 0.001). Intermediate UTS values were observed from the AMG group, which were not statistically different from control and Gly groups (*p* > 0.05), indicating partial inhibitory role of AMG. Neither the induced glycation nor the glycation inhibitor affected the energy to fracture. Ribose-induced glycation significantly increased the elastic modulus of dentine matrices. After 21 days ribose treatment, crown and root dentine showed a 1.9- and 1.3-fold stiffness increase, respectively, when compared to the control group. Regardless of the condition, the elastic modulus of dentine matrices increased after 21-day incubation (*p* = 0.023)

### The effect of AGEs on flexural strength, ultimate tensile strength and fracture resistance

Ultimate tensile strength, flexural strength and fracture resistance were assessed in two in vitro studies that evaluated the effects of AGEs or artificially induced glycation on non-diabetic dentine [39, 40]. Shinno et al. (2016) collected caries-free and fracture-free extracted teeth from patients of various ages and evaluated the relationship between the accumulation of AGEs in dentine through aging and the physical and mechanical properties of dentine. They evaluated the mineral density of crown dentine and measured the flexural strength of dentine using a three-point bending test and a universal testing machine. Toughness was calculated from the stress-strain curve of the flexural testing. They also measured the amount of AGEs in each specimen and correlate it to the flexural strength and toughness. They observed that aged dentine has a higher level of AGEs in its collagen, and that there is a significant negative correlation between the amount of AGEs and the flexural strength and toughness of crown and root dentine. The higher the levels of AGEs in dentinal collagen, the lower the flexural strength and toughness of the dentine [39]. Shinno et al. (2016) also observed increased mineral density and higher levels of occluded dentinal tubule lumens in aged dentine, which could further contribute to its decreased flexural strength. Microhardness and Young’s moduli of peritubular and intertubular dentine was also evaluated, although this did not change with aging [39].

In contrast, Alania et al. (2020) induced glycation of non-diabetic dentine with ribose solution and found that energy to fracture in artificially glycated dentine was not affected by the ribose-induced glycation, and the authors suggested that the lack of unidirectional collagen orientation in dentine may induce crack bridging and compensate for decrease in toughness of glycated dentine [40]. Alania et al. (2020) also found that ribose-induced glycation resulted in higher ultimate tensile strength and elastic modulus of dentine when compared with the control group [40]. They noted that the root dentine showed higher tensile strength and energy to fracture than the crown dentine and attributed the variation to the density and cross-linking of collagen in the root [40]. While Alania et al. (2020) and Shinno et al. (2016) did not specifically investigate the effect of diabetes on the mechanical strength of dentine, they met the inclusion criteria of this review as they investigated the effect of AGEs on the physical and mechanical properties of dentine. The level of AGEs is commonly elevated in diabetic patients [43] consequently, it is plausible to infer from their results that AGEs can affect the energy to fracture of dentine in diabetic patients.

### Effect of T2D on root fracture resistance

Two in vitro studies evaluated the vertical root fracture resistance (RFR) of T2D teeth [35, 37]. Both studies evaluated the RFR of premolars extracted from T2D patients. One study investigated the effect of different root canal obturation materialsincluding gutta-percha, white mineral trioxide aggregate (WMTA) and WMTA + Na_2_HPO_4_ on RFR in both T2D and non-T2D teeth [37]. They found that diabetic specimens with or without any obturation material have lower root fracture resistance compared with their normal non-diabetic counterparts [37].

Saghiri, et al. (2023) evaluated the effect of inorganic trace elements on RFR on T2D teeth and found that extracted teeth from T2D patients that were exposed to Magnesium (Mg), Strontium (Sr) and Zinc (Zn) had significantly increased resistance to root fracture [35]. Comparison between the results of RFR was not possible due to heterogeneity of methods between the two studies. Saghiri et al. (2021) carried out root canal treatment on the extracted teeth prior to RFR testing, while Saghiri et al. (2023) did not carry out any root canal treatment prior to RFR testing [35, 37]. This could explain why the RFR value of diabetic specimens without any intervention from Saghiri et al. (2023) was higher than that reported in Saghiri et al. (2021) even though both studies used the elastomeric material and acrylic resin to simulate periodontal ligament (PDL) and alveolar bone, respectively. The same vertical loads and speed were also used to induce root fracture in both studies.

### The effect of diabetes on shear, tensile and pushout bond strength

Limited studies have investigated the effect of diabetes on shear bond strength (SBS), tensile bond strength and pushout bond strength. The composite bond strength to T2D dentine has been shown to be related to T2D’s effects on dentinal tubule diameter and density [36, 38]. Universal adhesive (Scotchbond Universal) and total etch technique were used for all three studies that investigated the tensile bond strength and SBS of composite to dentine [36, 38, 41], while Attia et al. (2024) also investigated the shear bond strength using the self-etch technique [41]. The two studies that evaluated the SBS used crown dentine [36, 41], while the one study that evaluated the tensile bond strength used coronal root dentine [38]. The tensile bond strength of composite to dentine was observed to be significantly lower in T1D and T2D dentine than in controls, and T1D dentine also had significantly lower tensile bond strength than T2D dentine [38]. A significant difference in SBS has also been observed between non-diabetic and T1D dentine [36]. However, in the same study, no significant differences were observed between T1D and T2D dentine or between non-diabetic and T2D dentine [36]. In contrast, Attia et al. (2024) showed significant difference in SBS among non-diabetic, T1D and T2D groups. Their results showed non-diabetic specimens had significantly higher SBS compared with both T1D and T2D groups, and T2D had significantly higher SBS than T1D group [41]. They also found that SBS was higher when using the self-etch technique than the total-etch technique in all groups [41]. Due to differences in methodologies, the results between Attia et al. (2024) and Saghiri, Obeidi, et al. (2022) cannot be directly compared. In all three studies, the fracture pattern of non-diabetic specimens differed from diabetic specimens which exhibited more adhesive failure further suggesting hyperglycaemia may lead to weaker bond strength [36, 38, 41].

One study evaluated the push-out bond strength of WMTA packed into the root canals of single rooted extracted human teeth from diabetic and non-diabetic patients [33]. While the study did not specify whether T1D or T2D patients were included, it is assumed that the participants had T2D based on the study’s eligibility criteria. They found that dentine from T2D patients had significantly lower push-out bond strength of WMTA to root canal dentine compared to those without the disease [33]. They also observed structural failures in the diabetes group, indicating that failure may be due to either high tubular density in dentine or bond strength between WMTA to dentine is higher than the SBS [33]. However, their finding of increased tubular density in T2D dentine specimens contrasts with the results of an in vivo study on T1D rats, which found lower tubular density in T1D specimens compared with controls [31]. This difference may reflect differences in methodologies and the specimen type used.

The reduced SBS, tensile bond strength and push-out bond strength may be related to the observed increase in dentine tubular diameter and tubular density in diabetic dentine specimen [33, 36, 38]. Notably, tubular density has been observed to be significantly higher in diabetic dentine specimens [33].

### The effect of diabetes on the physical properties and trace element concentration of dentine

Diabetes has been shown to negatively affect the microhardness and concentration of trace elements within dentine [31]. In one of the two in vivo studies, diabetic induced rats had reduced dentine microhardness, and decreased intensity levels of Sr and Mg in the dentine [31]. Two other in vitro studies also investigated the effect of diabetes on human extracted premolars and evaluated the microhardness of dentine at 100- and 500- µm depths from the pulp-dentine interface [30, 35]. One study investigated the microhardness of mid-root dentine from extracted single rooted premolars between T2D and non-T2D patients, and compared the effects of chlorhexidine, NaOCl and EDTA on dentine microhardness and erosion susceptibility between both groups [30]. The researchers found that T2D as well as NaOCl and EDTA treatment reduced the surface microhardness of dentine significantly. Severe erosion was observed in some T2D specimens exposed to EDTA, but the difference was not statistically significant compared with the non-diabetic group [30]. In the second in vitro study, the roots of extracted human premolars from T2D patients were also examined for the effect of Mg, SR and Zn treatments on microhardness and tubular density of dentine [35] and the reported findings indicated that treatment with these trace elements significantly increased the microhardness of dentine and decreased dentine tubular density [35]. While the methodology between the two studies differed marginally in terms of sample preparation, the microhardness of diabetic specimens without any treatment was similar for both studies [30, 35]. However, in one of the studies, only one set of microhardness data was shown from the 100- and 500- µm depths, and it was unclear what depth was used for these measurements [30].

To further investigate the effect of diabetes on trace element concentrations in dentine, an in vitro study evaluated the difference in trace element levels of copper (Cu), lithium (Li), zinc, selenium (Se), strontium, manganese (Mn) and magnesium (Mg), while analyzing the nanostructure of T2D and non-T2D dentine [34]. They found significantly lower levels of Mg, Zn, Sr, Li, Mn and Se, and significantly higher levels of Cu in T2D specimens. T2D dentine also exhibited smaller crystallites and a less compact structure. The authors suggested that the differences in nanostructure and trace element concentration could play a role in the mechanical properties of dentine [34].

In a separate in vitro study the formation of AGEs in human dentinal collagen was explored and their potential impact on dentinal physiology was evaluated [29]. The study utilised six caries-free third molars of young and aged patients (young 18–26 yrs, aged 68–76 yrs). The teeth were demineralized for four weeks using a 10% EDTA solution, and some of the demineralized dentin samples were then subjected to in vitro glycation. They compared decalcified pulp-dentine specimens for the expression of AGEs in dentinal collagen and hardness of dentine from both the crown and the root of each tooth between both groups. Additionally, they also conducted the same tests on artificially glycated dentine from the young patients. Their results indicated that AGEs accumulate in dentinal collagen and levels are influenced by both a person’s age and physiological condition, such as the presence of hyperglycaemia. The hardness of the demineralized sections was assessed using a mechanical indentation tester. The hardness of aged dentine was higher than those obtained from young dentine, and the hardness of young dentine also increased after being artificially glycated [29]. Similar results have also been observed in two other in vitro study. Haluszka et al. (2022) and Alania et al. (2020) evaluated Young’s modulus and AGEs accumulation on artificially glycated dentine samples. In the ribose-treated samples, there was a higher level of AGE formation, and the length of the collagen fibers between dentinal tubules were also significantly greater than that found in the control group. The Young’s modulus in the ribose-treated dentine were also significantly higher than those in the control group [28, 40]. Notably, the stiffness of dentine increased with prolonged exposure to glycation [40] indicating that the accumulation of AGEs and glycation products can lead to a stiffening of dentine.

### The effect of diabetes on mineral density

In an in vivo study, Abbassy et al. (2015) evaluated the effect of T1D on the mineral density and morphological changes of dentine [27]. The study induced diabetes in 3-week old Wistar rats and found no significant differences in the effect of hyperglycaemia on the mineral density of enamel and dentine after 28 days, even though they observed a significant difference in the dentine mineral apposition and dentine formation rates. There was also a significant reduction in the thickness of dentine and enamel in diabetic samples [27]. The authors suggested that the elevated levels of blood glucose associated with T1D may impair the metabolic function of ameloblasts and odontoblasts, which may be similar to the mechanism by which T1D affects osteoblast function. Elevated blood glucose levels may also interfere with the mineralization of dentinal collagen matrix [27].

### The effect of rotary instrumentation on dentine from patients with diabetes

One in vitro study evaluated the amount of mid-root dentine removed by an endodontic rotary file in T1D, T2D and non-diabetic patients as a marker for the physical properties of dentine. The study recorded the amount of dentine removed by a common rotary endodontic instrument (ProTaper F3) and the depth of penetration of the file into dentine in a 120 s period. The findings indicated that more dentine was removed, and the rotary instrument penetrated more deeply in specimens from patients with T1- and T2D compared to non-diabetic specimens. T1D specimen have approximately a three times higher mean of depth of penetration than non-diabetic specimen, while T2D specimens had approximately two times higher mean depth of penetration. This suggests that hyperglycaemia may lead to structural changes that soften the dentine and the authors highlighted the translational significance of this including a risk of over-instrumentation and other procedural errors. However, further studies with better controlled factors, such as age and sex, should be conducted to corroborate these findings [32].

### Risk of bias evaluation

The risk of bias for animal studies were assessed using the SYRCLE tool [25] Table 3. For each study, a ‘Yes’, ‘No’, or ‘unclear’ was determined based on the item description. A low risk of bias was observed for baseline characteristics for both in vivo studies [27, 31]. Insufficient information was provided for allocation concealment, random housing of samples, intervention blinding, random outcome assessment and blinded outcome assessment. Saghiri et al. (2022) also had high risk of bias from selective outcomes reporting, sample size justification and other biases.

**Table 3:** Risk of bias for the in vivo studies included (SYRCLE)

Articles	Sequence generation	Baseline charcteristics	Allocation concealment	Random housing	Blinded interventions	Random outcome assessment	Blinded outcome assessment	Incomplete outcome data adequately addressed	free from selective outcome reporting	free of Other biases	Sample size justification
Saghiri, et al. (2022).	Unclear	Yes	Unclear	Unclear	Unclear	Unclear	Unclear.	No.	No	No	No
Abbassy et al. (2015)	Yes	Yes	Unclear	Unclear	Unclear	Unclear	Unclear.	Yes	Yes	Yes	No

Risk of bias assessment with in vitro studies was assessed using the modified Consolidated Standards of Reporting Trials (CONSORT) checklist [26]. A ‘Yes’ or ‘No’ was determined for each item description for each study (Table 4). Each study was given a final score of the overall risk of bias and was classified using the following system: Low (one or two item encoded as ‘No’), Medium (three to five items encoded as ‘No’), High (more than five items encoded as ‘No’). A high risk of bias was noted for two studies [28, 29], and a medium risk of bias was noted for four other studies [33, 39–41]. A high risk of bias was noted for generalizability for all studies, as these studies had relatively small sample sizes and in vitro conditions do not well reflect clinical conditions.

**Table 4:** Risk of Bias table for in vitro *studies* (CONSORT)

Articles	abstract	Eligibility criteria	Aim	Background	Objectives	Intervention	Outcomes	Sample Size	Statistical Method	Outcomes and estimation	Ancillary Analyses	Trial limitations	Generalizability	risk of bias
Miura et al. (2013)	Yes	No	Yes	Yes	No	Yes	yes	no	Yes	No	No	No	No	High
Haluszka (2022).	Yes	No	Yes	Yes	No	Yes	Yes	No	Yes	Yes,	No	No	No.	High
Alania et al. (2020).	Yes	No	Yes	Yes	Yes	Yes	Yes	No	yes	Yes	Yes	No	No	Medium
Attia et al. (2024)	Yes	Yes	Yes	Yes	Yes	Yes	Yes	No	Yes	Yes	Yes	No	No	Medium
Saghiri et al. (2020).	Yes	Yes	Yes	Yes	No	Yes	Yes	Yes	yes	Yes	No	No	No	Medium
Shinno et al., (2016).	No	Yes	Yes	Yes	No	Yes	Yes	No	Yes	Yes	Yes	No	No	Medium
Saghiri et al. (2022).	Yes	Yes	Yes	Yes	Yes	Yes	Yes	Yes	Yes	Yes	Yes	Yes	No	Low
Saghiri, Saghiri, et al. (2022)	Yes	Yes	Yes	Yes	Yes	Yes	Yes	Yes	Yes	Yes	Yes	Yes	No	Low
Saghiri et al. (2022)	Yes	Yes	Yes	Yes	Yes	Yes	Yes	Yes	Yes	Yes	Yes	Yes	No	Low
Saghiri et al. (2023).	Yes	Yes	Yes	Yes	Yes	Yes	Yes	Yes	Yes	Yes	Yes	Yes	No	Low
Saghiri et al. (2021).	Yes	Yes	Yes	Yes	Yes	Yes	Yes	yes	Yes	Yes	Yes	Yes	No	Low
Saghiri et al. (2021).	Yes	Yes	Yes	Yes	Yes	Yes	Yes	Yes	Yes	Yes	Yes	Yes	No	Low
Saghiri et al. (2022)	Yes	Yes	Yes	Yes	Yes	Yes	Yes	Yes	Yes	Yes	Yes	Yes	No	Low

## Discussion

This review evaluated the effects of diabetes and AGEs on the physical and mechanical properties of dentine. The results of the two in vivo studies suggest that diabetes has negative effects on dentine formation and mineral apposition, which in turn affects dentine’s microhardness, thickness and tubular density. Thirteen in vitro studies from human extracted teeth also showed significant differences between diabetes and non-diabetes teeth in terms of physical and mechanical properties. Most studies were in vitro, necessitating caution when interpreting findings to the clinic setting. It is important to note that factors such as donor age, tooth condition, and the time between diabetes diagnosis and sample collection may influence the results, in addition to whether the samples are from diabetic or non-diabetic patients.

This review comprehensively reports on relevant studies including those examining artificially induced glycation’s effects on dentine properties, providing a thorough summary, while acknowledging the limitations imposed by the diverse nature of the included studies. One of the limitations of the current review is that many studies were conducted by the same research group, which could potentially introduce researcher bias. The heterogeneity also complicates result comparisons, with outcomes skewed towards in vitro findings.

Diabetes impacts dentine development significantly; for instance, elevated glucose levels may inhibit odontoblast activity. Rat teeth affected by T1D had significantly lower total calcium level compared with those of their controls [44]. Furthermore, Abbassy et al., (2015) noted that hyperglycaemia could impair odontoblast metabolic function during tooth development, similar to osteoblasts’ calcium deposition inhibition in poorly controlled diabetes [27, 45]. As a result, the coronal dentine thickness decreased without significant differences in mineral density between diabetic and non-diabetic animals. This outcome is interesting and in contrast to the reduced bone mineral density associated with T2D [13, 46] and so further studies are needed to confirm these findings.

Elevated blood glucose levels associated with T1D may disrupt collagen matrix development and mineralization during dentine formation. A laboratory study using cultured human odontoblast-like cells indicated that high glucose concentrations significantly reduce Type 1 collagen production [47]. Collagen receives nutrients via the dentinal tubules and this is understood to regulate the mineral crystal architecture. Hence, this can be affected by hyperglycaemia which subsequently will affect the mechanical properties.

Glycation of collagen has been found to increase its Young’s modulus in two in vitro studies [28, 40], with both studies reporting that dentinal collagen becomes stiffer and fragile when it is cross-linked with AGEs reducing its viscoelasticity [28]. Immunohistochemical analyses have revealed that AGEs can accumulate in the collagen fibrils around the dentinal tubules [18, 29]. Cross-linking of collagen with AGEs can limit collagen deformation by restricting movement between collagen fibrils (fibril-fibril sliding) and inhibit unfolding or unwinding of individual collagen molecules [40].

**Fig. 2:**
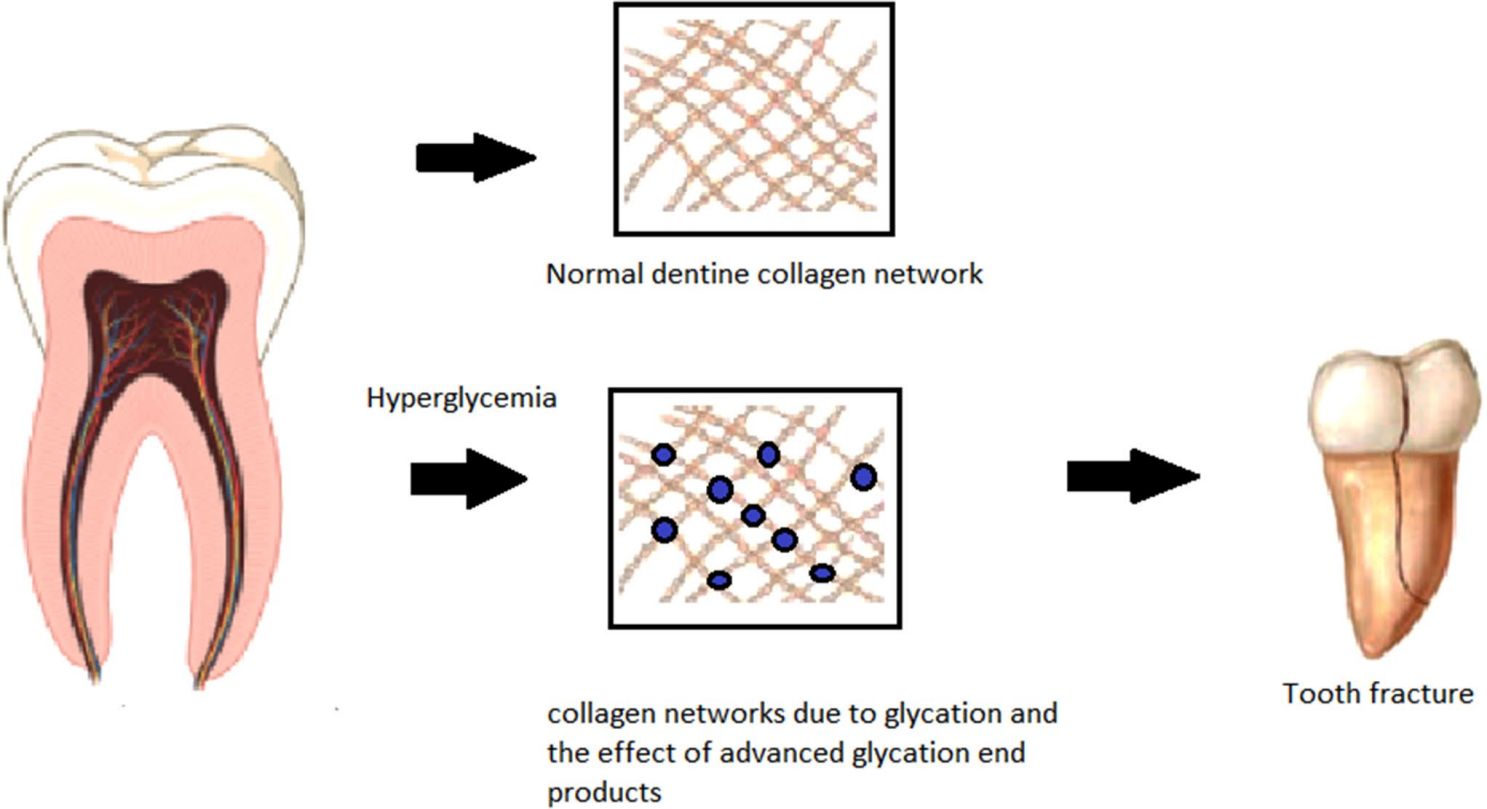
A diagram illustrating how AGE and other metabolites in diabetes lead to changes in dentine

Diabetes and hyperglycaemia were shown to decrease the root fracture resistance and microhardness of T2D teeth [37]. This reduction may stem from lower levels of trace elements like magnesium and strontium, which are critical for stabilizing calcium and stimulating dentinogenesis [44, 48]. The effects of magnesium on crystallinity are not entirely conclusive; it can destabilize hydroxyapatite structures by exchanging cations with calcium [31]. Disrupted calcium metabolism is commonly associated with T2D. When combined with physiological aging, AGEs, and occlusal factors, it is plausible that further deterioration in calcium levels may contribute to an increased risk of dentine cracks or fractures [49] as illustrated in Fig. 2. Only one study evaluated the effect of obturation materials on root fracture [37], and found the use of WMTA enhances fracture resistance. Further studies are needed to confirm this finding, and with advances in bonding technology, it would be valuable to further explore the potential impact of hyperglycaemia on the root fracture resistance.

Four in vitro studies and one in vivo study also observed a significant decrease in microhardness of dentine [29–31, 35, 39]. These changes may result from altered dentinal tubular structures, trace element reductions and AGE accumulation in collagen. AGEs can accumulate with age or conditions such as hyperglycaemia, making collagen harder and more brittle [29].

Changes in dentinal tubular diameter and density due to diabetes have been reported to affect push-out bond strength of MTA, composite shear and tensile bond strength in diabetic dentine [33, 36, 38]. T2D patients have been found to have higher dentinal tubule density and diameter, likely due to reduced mineralization of peritubular dentine and altered calcification of the tubules [33, 35]. This may explain the inverse relationship observed between the push-out bond strength of MTA to dentine and the tubular density [33, 35]. The shear and tensile bond strength of composite to dentine were also significantly lower between non-diabetic and both T1D and T2D dentine. Notably, conflicting results in shear bond strength between T1D and T2D dentine were found. While Saghiri et al. (2021) reported that there were no significant differences in shear bond strength between non-diabetic and T2D dentine, while Attia et al. (2024) found significant differences in shear bond strength between T1D and T2D dentine [36, 41]. Despite all three studies using 3 M Scotchbond Universal adhesives and total etch techniques, and with Attia et al. (2024) also exploring the self-etch technique, the differences in study design and methodologies may explain the varying findings [36, 38, 41]. More adhesive failures were also observed under scanning electron microscopy with the SBS test between resin composite and T1D dentine specimen, indicating an inferior bonding, while T2D dentine demonstrated mixed adhesive and cohesive failures [36, 41]. The reason for lower bond strength associated with diabetes may be due to decreased amounts of peritubular dentine [36]. However, the wide age range and small sample size of these studies limit the generalizability of the findings [36, 38, 41]. Future studies examining the effect of hyperglycaemia should consider age-matching participants and the effects of different bonding protocols as the outcomes have important translational significance including possible risks of coronal leakage from restorative failure or bonding failure between dentine and luting cements following fibre post placement.

Only one in vitro study has examined the effect of diabetes on root canal instrumentation using a novel experimental set up and found that significantly more dentine is removed from T1D and T2D dentine specimen than non-diabetic specimens [32]. This aligns with findings on reduced dentine microhardness, and highlights the increased risk of over instrumentation and canal transportation in diabetic patients.

Most of the in vitro studies in this review have relatively small sample sizes from a wide age range of patients and there were differences in methodologies used. Indeed, physiological aging is associated with an increased level of AGE accumulation and there may be gender differences [50]. Sclerotic dentine formation can influence the mechanical and physical properties of dentine. Furthermore, moisture impacts dentine’s physical properties, however it is unclear whether dry or wet samples were used in all studies. In a hydrated environment, dentine’s elastic modulus can decrease by 35%, and hardness by 30% [51]. With regards to the SBS and tensile bond strengths tests conducted, none of the two in vitro studies used thermocycling or dynamic loading of the specimens to simulate the oral environment, which limit the clinical generalizability of the findings [36, 41]. The blade method was also used to apply the shear stress [36]. However, it has shown that having the crosshead blade closer to the substrate which can effectively reduce the tensile part such as enclose-mould method [52] or Ultradent Shear bond strength test method in ISO 29,022 [53].

The prognosis of root canal treatment depends on various pre-operative, intra-operative and post-operative factors. Systematic review and meta-analysis indicate that diabetes should be considered as an important pre-operative prognostic factor in root canal treatment. A recent meta-analysis revealed a significant association between diabetes and the frequency of root filled teeth with radiographic signs of persistent periapical disease [54]. Diabetes patients are also two and a half times more likely to lose root filled teeth [55] and the risk of vertical root fracture is high [20]. While the biological mechanisms by which T2D can result in a greater loss of teeth is not well understood, previous research has observed changes in the pulp and dentine of patients with T2D [17, 18, 30, 33]. Consequently, increased knowledge in this area is required to assist dentists in treatment planning and the consent processes with these patients.

## Conclusion

1. DM and AGEs negatively influence the physical and mechanical properties of dentine however, there is a paucity of evidence with further studies being needed.
2. There is a heterogeneity of studies investigating the effect of physical and mechanical properties of dentine and questions remain around the true impact of systemic disease on dental hard tissue.
3. While not yet explicitly clear, there is a growing body of evidence indicating that DM may lead to changes in the dentine similar to hard tissues in other body sites and this is could negatively influence dentine bonding and resistance to fracture. Clinicians should carefully consider a patients systemic health status when planning restorative and endodontic treatment.

## Data Availability

No datasets were generated or analysed during the current study.
